# Aspirin Eugenol Ester Alleviates Vascular Endothelial Ferroptosis by Enhancing Antioxidant Ability and Inhibiting the JNK/c-Jun/NCOA4/FTH Signaling Pathway

**DOI:** 10.3390/antiox14101220

**Published:** 2025-10-10

**Authors:** Ji Feng, Qi Tao, Zhi-Jie Zhang, Qin-Fang Yu, Ya-Jun Yang, Jian-Yong Li

**Affiliations:** Key Lab of New Animal Drug of Gansu Province, Key Lab of Veterinary Pharmaceutical Development of Ministry of Agriculture and Rural Affairs, Lanzhou Institute of Husbandry and Pharmaceutical Sciences of CAAS, Lanzhou 730050, China; 82101235821@caas.cn (J.F.); taoqi19951224@163.com (Q.T.); 18793107830@163.com (Z.-J.Z.);

**Keywords:** aspirin eugenol ester (AEE), transcriptomics, ferroptosis, oxidative stress, JNK/c-Jun

## Abstract

Oxidative stress occurs within bovine when exposed to harmful stimuli, accompanied by substantial accumulation of reactive oxygen species. Without timely clearance, these reactive oxygen species attack vascular endothelial cells, concurrently inducing extensive production of lipid peroxides within the vascular endothelium, and thereby triggering ferroptosis. Aspirin eugenol ester (AEE) showed pharmacological activity against oxidative stress-induced vascular endothelial damage. However, whether it could alleviate vascular endothelial damage by inhibiting ferroptosis remains unclear. This study aimed to evaluate the effects of AEE on vascular endothelial ferroptosis and elucidate its underlying molecular mechanisms. This study established vascular endothelial damage models in vitro and in vivo to explore the ability of AEE to inhibit ferroptosis and oxidative stress by measuring ferroptosis- and oxidative stress-related biomarkers. Transcriptomic and network pharmacology analyses were performed to identify AEE-regulated pathways and key targets. Validation of the pathways were conducted using molecular docking, cellular thermal shift assay, and specific protein agonists/inhibitors. AEE inhibited oxidative stress and ferroptosis in bovine aortic endothelial cells induced by hydrogen peroxide (H_2_O_2_) or RSL3 via suppressing the upregulation of ferroptosis-related genes and enhancing the expression of antioxidant genes. Transcriptomic and network pharmacology analyses identified JNK as a core target of AEE in regulating ferroptosis. JNK agonists enhanced H_2_O_2_-induced ferritinophagy; on the contrary, JNK inhibitors alleviated it. AEE suppressed H_2_O_2_-induced phosphorylation of JNK/c-Jun and ferritinophagy. In a carrageenan-induced rat aortic vascular endothelial damage model, AEE alleviated vascular endothelial damage and ferroptosis-related gene changes, promoted antioxidant gene expression, and inhibited JNK/c-Jun phosphorylation and ferritinophagy. AEE inhibited vascular endothelial ferroptosis by enhancing antioxidant ability, blocking downstream ferritinophagy, and reducing ferrous ion release.

## 1. Introduction

Cattle breeding is a core sector within husbandry. Effective health management interventions for cattle constitute an essential mechanism for reducing bovine morbidity rates while enhancing productive performance. Vascular endothelial cells (VECs) play a pivotal role in bovine productivity and immune defense systems, directly orchestrating host defense mechanisms against invasive microbial pathogens [1]. VECs establish protective physical barriers to prevent pathogenic bacteria from invading the basement tissue. Simultaneously, VECs also modulate multiple homeostatic processes, including vascular tone regulation, microvascular permeability modulation, hemodynamic equilibrium maintenance, and adaptive immune response coordination [2]. 

VECs dysfunction induce a spectrum of bovine pathologies, including thrombosis, atherosclerosis, and mastitis [3,4,5]. Endothelial damage primarily arises from disruption of redox homeostasis and immune functional equilibrium. Subsequently, metabolic dyshomeostasis induces mitochondrial electron transport chain dysfunction, resulting in excessive accumulation of reactive oxygen species (ROS), which is a phenomenon that is particularly prominent in tissues with high mitochondrial metabolic activity, such as VECs and others [6,7]. Accumulated ROS in VECs not only directly attack the VEC membrane but also inhibit the activity of antioxidant enzymes—including superoxide dismutase (SOD), glutathione peroxidase 4 (GPX4), catalase, and glutathione peroxidase—while inducing the production of large amounts of lipid peroxides. This process triggers ferroptosis [8,9,10]. Ferroptosis is an iron-dependent form of regulated cell death and serves as an important mechanism underlying ROS-induced VECs death. It differs from ROS-induced apoptosis and necrosis, characterized by a pathological iron accumulation-induced lipid peroxidation cascade, ultimately leading to plasma membrane rupture and cell death [11,12]. The core molecular events include the following: under oxidative stress stimulation, nuclear receptor coactivator 4’s (NCOA4) expression increased, specifically bound ferritin heavy chain (FTH), mediating its lysosomal translocation to trigger ferroptosis via ferritinophagy, which caused an intracellular free ferrous ion (Fe^2+^) overload [13,14,15]. Excessive Fe^2+^ catalyze hydroxyl radical (·OH) generation via the Fenton reaction subsequently attacks membrane polyunsaturated fatty acids (PUFAs) to initiate lipid peroxidation chain reactions [14]. In this process, ROS exhibits dual regulatory roles: (1) ROS enhances NCOA4 expression to promote ferritin degradation and Fe^2+^ release, thereby amplifying ROS production; (2) ROS facilitates Fenton reaction kinetics through oxidizing microenvironments [16]. The synergistic interaction between the NCOA4-FTH axis-driven ferrous dysregulation and ROS hyperactivity drives catastrophic lipid peroxidation, culminating in plasma membrane integrity disruption and irreversible activation of cell death execution programs. Current studies have demonstrated that ferroptosis in VECs contributes to vascular aging, vascular dysfunction, and vascular calcification, serving as a critical factor in the progression of diseases such as hypertension, thrombosis, and atherosclerosis [17,18,19,20]. Given the pivotal role of ferroptosis in VECs across various pathologies, countermeasures to mitigate ferroptosis in vascular endothelial cells require further exploration and development.

Aspirin eugenol ester (AEE), a novel ester compound synthesized via esterification reaction, features a molecular architecture formed through dehydration condensation between the carboxylic acid group of aspirin and the phenolic hydroxyl group of eugenol. Pharmacological studies have demonstrated that AEE has exhibited multifaceted pharmacological activity, including anti-inflammatory effects, antioxidant stress capacity, inhibition of platelet aggregation, and prevention of thrombosis [21,22]. Its antioxidant stress effects have been validated in multiple experimental models: (1) In a hydrogen peroxide (H_2_O_2_)-induced oxidative stress model, using human umbilical vein endothelial cells (HUVECs), AEE-elevated reduced glutathione (GSH) levels, and SOD activity, while reducing malondialdehyde (MDA) content, thereby significantly attenuating apoptosis and oxidative damage [23]. (2) Similarly, dose-dependent protective effects of AEE have been observed in both paraquat-induced oxidative damage models of human alveolar epithelial cells (A549) and rat pulmonary tissues [24]. Notably, ferroptosis exhibited significant mechanistic crosstalk with oxidative stress pathways. However, current research remained devoid of investigations into the modulatory effects of AEE on ferroptosis signaling pathways, and left its regulatory mechanisms and therapeutic potential unexplored.

Given the strong connection between oxidative stress and ferroptosis, it is speculated that AEE may attenuate ferroptosis induced by oxidative stress. This study systematically evaluated the effects of AEE on oxidative stress-induced ferroptosis in bovine aortic endothelial cells (BAECs), providing preliminary insights into its underlying mechanisms to provide novel theoretical insights for developing therapeutic strategies against bovine vascular endothelial damage.

## 2. Materials and Methods

### 2.1. Reagents and Antibodies

AEE (LOT:20190701, purity ≥ 99.6%) was chemically synthesized by the Lanzhou Institute of Husbandry and Pharmaceutical Sciences of CAAS; Ferrostatin-1 (Fer-1) (HY-100579), RSL3 (HY-100218A), SP600125 (HY-12041), and Anisomycin (HY-18982) were purchased from MedChemExpress (Shanghai, China). A 3% H_2_O_2_ solution (323381), Carrageenan (κ-Car) (C1013), and Urethane (U2500) were purchased from Sigma-Aldrich (St. Louis, MO, USA). Sodium carboxymethyl cellulose (CMC-Na) (30036328) was purchased from Sinopharm Chemical Reagent Co., Ltd. (Shanghai, China). Fetal Bovine Serum (AUS-01E-02) was purchased from Cell-Box (Changsha, China). RPMI-1640 (11875093) and 0.25% trypsin (25200072) were purchased from Gibco (Grand Island, NE, USA). NCM Western Blot Stripping Buffer (WB6200), NCM Universal Antibody Diluent (WB100D), NcmBlot blocking buffer (P30500) and Cell Counting Kit-8 (C6050) were purchased from NCM Biotech (Suzhou, China). Protease and phosphatase inhibitor cocktail for general use (P1045), LDH Cytotoxicity Assay Kit with WST-8 (C0018S), Lipid Peroxidation Assay Kit with BODIPY 581/591 C11 (S0043S), DAPI Staining Solution (C1006), Immunol Staining Fix Solution (P0098), Immunol Staining Blocking Buffer (P0102), and Enhanced Immunostaining Permeabilization Buffer (P0097) were purchased from Beyotime (Shanghai, China). SDS-PAGE loading buffer, 5× (with DTT) (P1040), RIPA buffer (high) (R0010), TriQuick Reagent (R1100), and Reduced Glutathione (GSH) Content Assay Kit (BC1175) were purchased from Solarbio (Beijing, China). Rat Vascular Cell Adhesion Molecule 1 (VCAM1) ELISA Kit (JL11762), Rat Tumor Necrosis Factor Alpha (TNF-α) ELISA Kit (JL13202), Rat Interleukin 6 (IL-6) ELISA Kit (JL20896), and Rat Endothelin 1 (ET-1) ELISA Kit (JL10931) were purchased from Jianglai Biotechnology (Shanghai, China). Liquid Sample Malondialdehyde (MDA) Assay Kit (TBA method) (E2019) and Ferrous Ion Content Assay Kit (E1046) were purchased from Applygen Technologies Inc. (Beijing, China). PrimeScript™ RT Master Mix (Perfect Real Time) (RR036Q), TaKaRa MiniBEST Universal RNA Extraction Kit (9767), and TB Green^®^ Premix Ex Taq™ II FAST qPCR (CN830A) were purchased from Takara Biomedical Technology (Beijing) Co., Ltd. (Beijing, China). XPAGE™ (X12412Gel) was purchased from AEC Biotechnology (Changzhou, China). JNK (ab179461, 1:1000 for Western blot), p-JNK (ab76572, 1:5000 for Western blot), c-Jun (ab40766, 1:5000 for Western blot), c-Jun (phospho S63) (ab32385, 1:1000 for Western blot), NCOA4 (ab222071, 1:300 for Immunofluorescence), FTH (ab75973, 1:1000 for Western blot, 1:200 for Immunofluorescence), β-actin (ab8227, 1:5000 for Western blot), Goat Anti-Rabbit IgG H&L (HRP) (ab6721, 1:5000 for Western blot), and Goat Anti-Rabbit IgG H&L (Alexa Fluor^®^ 488) (ab150077, 1:1000 for Immunofluorescence) were purchased from Abcam (Cambridge, UK).

### 2.2. Animal Experiments

Wistar rats were sourced from the Experimental Animal Center of Lanzhou Veterinary Research Institute of CAAS. Male rats aged 8–10 weeks (body weight 200 ± 20 g) were selected for this study. All the rats were housed in specific pathogen-free (SPF) barrier facilities. All experimental protocols and procedures were approved by the Institutional Animal Care and Use Committee of Lanzhou Institute of Husbandry and Pharmaceutical Science of the Chinese Academy of Agricultural Sciences (Approval No. NKMYD202510; Approval Date: 24 March 2025). Animal welfare and experimental procedures were performed strictly using the Guidelines for the Care and Use of Laboratory Animals, issued by the US National Institutes of Health. Fifty rats were randomly assigned into 5 groups (*n* = 10): Control group (Control), κ-Car group (κ-Car), AEE low-dose group (AEE-L), AEE medium-dose group (AEE-M), and AEE high-dose group (AEE-H). AEE was prepared as a suspension using 0.5% CMC-Na, and κ-Car was prepared in saline as an injectable solution. The AEE-L, AEE-M, and AEE-H groups received 9, 18, and 36 mg/kg/day AEE via oral gavage, respectively, while the Control and κ-Car groups were administered equivalent volumes of 0.5% CMC-Na. On day 7 of AEE administration, intraperitoneal injections of κ-Car (20 mg/kg) were administered to the κ-Car and AEE groups, while the Control group received equivalent volumes of saline. On day 8, rats were anesthetized with urethane, and blood was collected via abdominal aortic puncture. Plasma and aortic tissues were harvested for subsequent biochemical and histopathological analyses.

### 2.3. Hematoxylin–Eosin (HE) Staining

The rat aortas were fixed in a 4% paraformaldehyde solution for 24 h. Subsequently, the tissues were paraffin-embedded and sectioned into 5 μm thick slices. After dewaxing, the sections underwent hematoxylin and eosin staining and were examined under a light microscope.

### 2.4. Immunohistochemistry

The rat aortas were fixed in a 4% paraformaldehyde solution for 24 h. Subsequently, the tissues were paraffin-embedded and sectioned into 5 μm-thick slices. After dewaxing, antigen retrieval was performed using sodium citrate solution. The sections were incubated with corresponding primary and secondary antibodies and examined under a light microscope.

### 2.5. Enzyme-Linked Immunosorbent Assay (ELISA)

The levels of IL-6, TNF-α, VCAM-1, and ET-1 in rat plasma and aortic tissues were measured following the manufacturer’s instructions for the ELISA kit. Plasma samples were measured directly. Tissues were rinsed and ground with PBS, and the resulting homogenate was centrifuged at 5000× *g* for 5–10 min; the supernatant was collected for detection. For the assay, 100 μL of sample or standard solutions were added to a pre-coated assay plate and incubated at 37 °C for 60 min. The liquid was then discarded, and 100 μL of biotin-antibody working solution was added, followed by incubation at 37 °C for 60 min. After discarding the liquid, the plate was washed three times. Next, 100 μL of streptavidin-HRP working solution was added and incubated at 37 °C for 30 min, followed by five washes. Subsequently, 90 μL of tetramethylbenzidine substrate was added, and it was incubated at 37 °C in the dark for 15 min. Finally, 50 μL of stop solution was added to terminate the reaction. We measured the absorbance at 450 nm and calculated the level of IL-6, TNF-α, VCAM-1, and ET-1.

### 2.6. Cell Culture

The BAEC line was provided by Guangzhou Jennio Biotech Co., Ltd. (Guangzhou, China). Cells were cultured in RPMI-1640 complete medium supplemented with 10% fetal bovine serum (FBS) and maintained in a humidified incubator at 37 °C with 5% CO_2_. The culture medium was replaced every 48 h. When cell confluency reached >90%, BAECs were dissociated using trypsin. Cell passage numbers were controlled between 5 and 10 generations.

### 2.7. Cell Viability Assay

BAECs in the logarithmic growth phase with healthy growth status were seeded into 96-well culture plates at a density of 1 × 10^5^ cells/mL. After 24 h, cells were treated with drug-containing media at varying concentrations for different durations. Following drug treatment, the supernatant was aspirated, and 100 μL of CCK-8 detection working solution (prepared at a 1:9 ratio of CCK8 to culture medium) was added to each well. Plates were incubated at 37 °C for 2 h, and cell viability was calculated by measuring absorbance at 450 nm.

### 2.8. Cell Experiment Design

BAEC were seeded at 1 × 10^5^ cells/mL in culture flasks or plates and cultured for 48 h. After, damage models were established by treating cells with 500 μM H_2_O_2_ [25] for 24 h or 20 μM RSL3 [26] for 6 h. Pretreatment with AEE (4, 8, 16 μM), ferroptosis inhibitor Fer-1 (40 μM), JNK inhibitor SP600125 (2.5 μM), or JNK agonist Anisomycin (250 nM) was performed 24 h before damage model induction.

### 2.9. Lactate Dehydrogenase (LDH) Release Rate Assay

We followed the manufacturer’s instructions for the LDH assay kit to process cell samples. We centrifuged the supernatants at 800 rpm for 5 min. We transferred 100 μL of the centrifuged supernatant to new 96-well culture plates. We added detection solution and incubated the samples at 25 °C in the dark for 25 min. Then, we measured the absorbance at 450 nm and calculated LDH release rates.

### 2.10. GSH Level Measurement

Cell samples were washed twice with PBS, and 400 μL of Reagent 1 was added according to the instructions of the Reduced Glutathione (GSH) Content Assay Kit (BC1175). Cell lysis was collected via freezing and thawing three times with liquid nitrogen, then centrifuged at 12,000× *g* for 4 min at 4 °C to obtain the supernatant for measurement. Plasma samples were directly assayed. For the GSH measurement, 20 μL of the cell supernatant or plasma sample, followed by 140 μL of Reagent 2 and 40 μL of Reagent 3, were added to a 96-well plate. The mixture was mixed well and incubated at room temperature for 2 min. Subsequently, absorbance was measured at 412 nm to calculate the GSH level in each sample.

### 2.11. MDA Level Measurement

Cell samples were washed twice with PBS, and 200 μL of extraction solution was added according to the instructions of the Liquid Sample Malondialdehyde (MDA) Assay Kit (TBA method) (E2019). Cell lysis was collected via ultrasonication, then centrifuged at 10,000× *g* for 10 min at 4 °C to obtain the supernatant for measurement. Plasma samples were directly assayed. Tissues were homogenized in 500 μL of extraction solution and centrifuged at 10,000× *g* for 10 min at 4 °C to obtain the supernatant for measurement. For the MDA measurement, 100 μL of the sample was added to a 1.5 mL centrifuge tube, followed by 100 μL of thiobarbituric acid solution. The mixture was mixed well and heated at 95 °C for 30 min, then cooled on ice to room temperature. After centrifugation at 10,000× *g* for 10 min, 200 μL of the supernatant was collected to a 96-well plate, and absorbance was measured at 535 nm to calculate the MDA level in each sample.

### 2.12. Fe^2+^ Level Measurement

Cell samples were washed twice with PBS, and 300 μL of extraction solution was added according to the instructions of the Ferrous Ion Content Assay Kit (E1046). Cell lysis was collected via ultrasonication, then centrifuged at 10,000× *g* for 10 min at 4 °C to obtain the supernatant for measurement. Tissues were homogenized in 500 μL of extraction solution and centrifuged at 10,000× *g* for 10 min at 4 °C to obtain the supernatant for measurement. For the Fe^2+^ measurement, 200 μL of the sample was added to a 1.5 mL centrifuge tube, followed by 100 μL of colorimetric working solution. The mixture was mixed well and incubated at 37 °C for 10 min. Subsequently, 200 μL of the mixture was transferred to a 96-well plate, and absorbance was measured at 593 nm to calculate the Fe^2+^ level in each sample.

### 2.13. Lipid Peroxide (LPO) Level Measurement

Cell samples were washed once with PBS and treated with 37 °C BODIPY 581/591 C11 working solution for 10 min, following the manufacturer’s instructions. Then, cells were washed twice with PBS. Cells were visualized under a fluorescence microscope. BODIPY 581/591 C11 exhibited maximum excitation/emission wavelengths of 581/591 nm in its reduced state, which shifted to 488/510 nm upon oxidation by lipid hydroperoxides. The red fluorescence was observed in the reduced state, the green fluorescence was observed in the oxidized state, and the red-to-green fluorescence ratio was used to determine the degree of lipid peroxidation.

### 2.14. RT-qPCR

The total RNA of cell and artery tissues was extracted with the RNA extraction kit. RNA concentrations were quantified using a NanoDrop spectrophotometer (Thermo Fisher Scientific, Waltham, MA, USA). The cDNA was synthesized by using a reverse transcription kit. qPCR was performed using a fast qPCR kit. The qPCR program was as follows: in the pre-denaturation step, 95 °C for 30 s; and in the PCR step, 95 °C for 5 s, and 60 °C for 10 s, for a total of 40 cycles. GAPDH served as the endogenous control for cell samples, while β-actin was used for tissues. Relative gene expression levels were calculated using the 2^−ΔΔCt^ method. Primer sequences are listed in Table 1.

### 2.15. Transcriptome Analysis

BAECs were seeded at 1 × 10^5^ cells/mL in 75 cm^2^ culture flasks and cultured for 24 h. Subsequently, the H_2_O_2_ model group and the 8 μM AEE administration group were established and treated according to the previously described protocol. Total RNA was extracted from the cells after treatment, and a bipartite 150 bp sequencing library was constructed and high-throughput sequenced using the Illumina NovaSeq X Plus platform. Differential expression analysis between the H_2_O_2_ and AEE groups was performed using DESeq2 (version 1.40.2). Differentially expressed genes (DEGs) were defined as *p* < 0.05 & |log2FC| ≥ 1. Gene ontology (GO) enrichment analysis was performed using Goatools (version 1.3.2) with Fisher’s exact test. Kyoto Encyclopedia of Genes and Genomes (KEGG) enrichment analysis was performed using the Python SciPy package 1.6.0 with Fisher’s exact test. GO terms and KEGG pathway with *p* < 0.05 were considered statistically significantly enriched.

### 2.16. Network Pharmacology Analysis

Swiss Target Prediction (http://www.swisstargetprediction.ch/, accessed on 24 January 2025) [27] was used to predict the AEE protein target. The keyword “Ferroptosis” was searched in OMIM (https://www.omim.org/, accessed on 24 January 2025) [28], GeneCards (https://www.genecards.org/, accessed on 24 January 2025) [29] and FerrDb (https://www.zhounan.org/ferrdb/current/, accessed on 24 January 2025) [30]. Following the merging and deduplication of the results from the three databases, ferroptosis-associated targets were obtained. The Venn network graph was constructed using the Venn online tool (http://bioinformatics.psb.ugent.be/webtools/Venn/, accessed on 24 January 2025) to obtain the intersecting targets of AEE and ferroptosis. The STRING database (https://string-db.org/, accessed on 24 January 2025) [31] was used to construct and analyze protein–protein interaction (PPI) networks of intersecting targets. The PPI network was imported into Cytoscape 3.10.3 [32] for visualization. The CytoHubba plugin was used to analyze the topological properties of each node in the network.

### 2.17. Molecular Docking

The crystal structures of JNK were obtained from the Protein Data Bank (PDB) (https://www.rcsb.org/, accessed on 26 January 2025). PyMOL 2.5.0 [33] and AutoDock Tool 1.5.7 were used to preprocess the structures of JNK, including hydrogenation, dehydration, and other pretreatments. Docking sites and grid boxes were defined based on the original ligand positions and relevant references. The binding affinity between JNK and AEE was evaluated via molecular docking with AutoDock Vina 1.2.5 [34]. Visualization of the docking results was performed with PyMOL 2.5.0 and Discovery Studio 2019 Client software.

### 2.18. Immunofluorescence Analysis

BAECs were seeded in a 12-well plate with coverslips (1 × 10^5^ cells/well). After the drug treatment was finished, the BAECs were fixed with Immunol Staining Fix Solution for 15 min, permeabilized with Enhanced Immunostaining Permeabilization Buffer for 15 min, and blocked with Immunol Staining Blocking Buffer for 15 min. Then, 300 μL of the primary antibody was added to each well and they were incubated at 4 °C for 24 h, and incubated with the secondary antibody at 25 °C for 2 h, followed by DAPI staining for 5 min. Fluorescence was detected with a fluorescence microscope.

### 2.19. Cellular Thermal Shift Assay (CETSA)

BAECs were seeded at 1 × 10^5^ cells/mL in 75 cm^2^ culture flasks and cultured for 24 h. Subsequently, the cells were treated with a complete culture medium at a concentration of 8 μM AEE or 0.1% DMSO for 24 h. After washing twice with PBS, cell samples were collected and resuspended in PBS. The resuspension was aliquoted into centrifuge tubes at 50 μL, treated at a designated temperature for 3 min, incubated at room temperature for 3 min, and frozen/thawed three times with liquid nitrogen. The cell lysate was centrifuged at 20,000× *g* for 20 min at 4 °C to obtain the supernatant for Western blotting.

### 2.20. Western Blot

Cell samples were washed twice with PBS, while tissue samples were quickly frozen in liquid nitrogen and ground. The processed samples were lysed with RIPA containing protease and phosphatase inhibitors for 30 min at 4 °C. Lysates were centrifuged at 12,000× *g* for 5 min at 4 °C to obtain the supernatant. The supernatant of proteins was mixed with SDS-PAGE loading buffer and denatured by boiling at 100 °C for 10 min.

Each protein sample (15 μg) was loaded into an SDS-PAGE gel (4–20%) for separating protein using a constant voltage of 150 V for 55 min. Subsequently, proteins were transferred to 0.45 μM PVDF membranes at a constant current of 400 mA for 35 min. The PVDF membrane was blocked with a blocking buffer for 10 min and incubated with primary antibodies overnight at 4 °C. The membranes were then incubated with the secondary antibody at room temperature for 2 h. The bands of proteins were detected under the chemiluminescence imaging system.

The band intensities of CETSA were normalized by setting the protein intensity retained in the supernatant at 42 °C as 100%, with subsequent bands expressed as percentages. For all other samples, the relative expression levels of target proteins were calculated as the ratio of target protein intensity to reference protein intensity.

### 2.21. Statistical Analysis

Statistical analyses and graphing were performed using the one-way ANOVA and Tukey’s multiple comparisons test of GraphPad Prism 10.1.2 software. Data are represented as mean ± SD. A significance level of *p* < 0.05 was considered statistically significant.

## 3. Results

### 3.1. AEE Attenuates H_2_O_2_ or RSL3-Induced BAEC Damage

**Figure 1:**
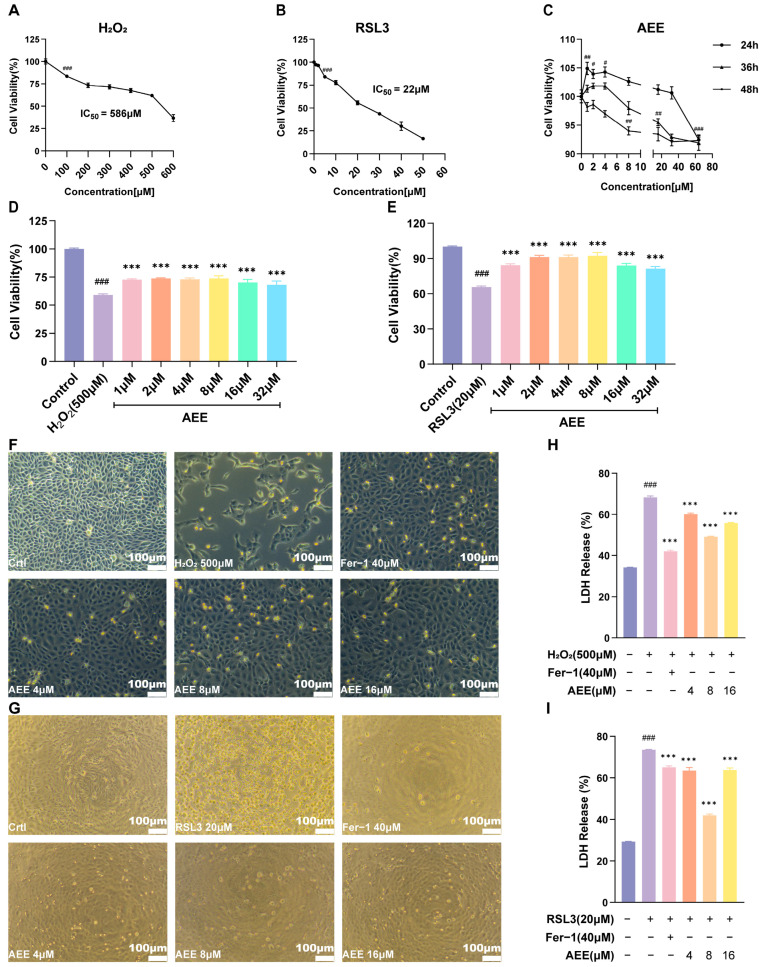
AEE attenuated H_2_O_2_ or RSL3-induced BAECs damage. (**A**) Cell viability of BAECs treated with different concentrations of H_2_O_2_ for 24 h (*n* = 6). (**B**) Cell viability of BAECs treated with different concentrations of RSL3 for 6 h (*n* = 6). (**C**) Cell viability of BAECs treated with different concentrations of AEE for 24 h, 36 h, and 48 h (*n* = 6). (**D**,**E**) Effect of different concentrations of AEE pretreatment on the cell viability of H_2_O_2_ or RSL3- induced BAECs (*n* = 6). (**F**,**G**) Effects of AEE and Fer-1 pretreatment on H_2_O_2_ or RSL3-induced BAECs damage under 10× light microscopy, (Scale bar = 100 μm). (**H**,**I**) Effect of AEE pretreatment on the LDH release of H_2_O_2_ or RSL3-induced BAECs (*n* = 3). Data are represented as mean ± SD. # *p* < 0.05, ## *p* < 0.01, ### *p* < 0.001 versus the Control group; *** *p* < 0.001 versus the H_2_O_2_ or RSL3 group.

From Figure 1A, at the concentration range of 100–600 μM, H_2_O_2_ significantly reduced BAEC cell viability in a concentration-dependent manner. (*p* < 0.05). When the concentration of H_2_O_2_ reached 500 μM, BAEC cell viability decreased to 61.8%, and the IC_50_ = 586 μM. As shown in Figure 1B, at the concentration range of 5–50 μM, RSL3 significantly reduced BAEC cell viability in a concentration-dependent manner (*p* < 0.05). When the concentration of RSL3 reached 20 μM, BAECs cell viability decreased to 55.6%, and the IC_50_ = 22 μM. Fer-1 was not significantly toxic to BAEC in the selected concentration range (Figure S1). At all concentrations of AEE and different treatment durations, BAEC cell viability remained above 90% (Figure 1C). As treatment time increased, cell viability in each AEE concentration group showed a decreasing trend. Specifically, after 24 h treatment with AEE at 1–4 μM, cell viability was significantly higher than that in the Control group. After 24 h treatment with AEE at 8–32 μM, cell viability was not significantly different from that in the Control group. After 24 h treatment with AEE at 64 μM, cell viability was significantly lower than that in the Control group. For AEE at 1–8 μM treated for 36 h, cell viability was not significantly different from than that in the Control group. After 36 h treatment with AEE at 16–64 μM, cell viability was significantly lower than that in the Control group. However, after 48 h treatment with AEE at 8–64 μM, cell viability was significantly lower than that in the Control group. Therefore, 500 μM H_2_O_2_ and 20 μM RSL3 were selected to establish the BAECs damage model, with 1–32 μM as the AEE pretreatment concentration and 24 h as the AEE pretreatment duration.

From Figure 1D,E, BAECs cell viability in the H_2_O_2_ group decreased to 59% (*p* < 0.05). In contrast, BAEC cell viability in all AEE groups was significantly higher than that in the H_2_O_2_ group, showing a trend of first increasing and then decreasing. The highest cell viability (73.6%) was observed in the 8 μM AEE group (*p* < 0.05). BAECs cell viability in the RSL3 group decreased to 65.5% (*p* < 0.05). In contrast, BAEC cell viability in all AEE groups was significantly higher than that in the RSL3 group, showing a trend of first increasing and then decreasing. The highest cell viability (92.2%) was observed in the 8 μM AEE group (*p* < 0.05). Therefore, 4, 8, and 16 μM were further selected as concentrations of AEE pretreatment in subsequent experiments.

From Figure 1F,H, compared with the Control group, H_2_O_2_ induced significant morphological changes in BAECs, including nuclear fragmentation, cell membrane rupture, blurred cell boundaries, extensive cell death and detachment, and a marked increase in the LDH release rate (up to 68.1%). AEE significantly alleviated H_2_O_2_-induced morphological changes, cell death, and LDH release elevation in BAECs (*p* < 0.05). Similarly, pretreatment with the ferroptosis inhibitor Fer-1 also alleviated cell death, detachment, and LDH release-rate elevation (*p* < 0.05). RSL3 induced the shrinkage and rounding of cells and extensive cell detachment, with a significant increase in the LDH release rate in BAECs (Figure 1G,I). AEE or Fer-1 inhibited RSL3-induced morphological changes, cell detachment, and LDH-release elevation (*p* < 0.05).

### 3.2. AEE Inhibited H_2_O_2_ or RSL3-Induced Ferroptosis in BAECs

**Figure 2:**
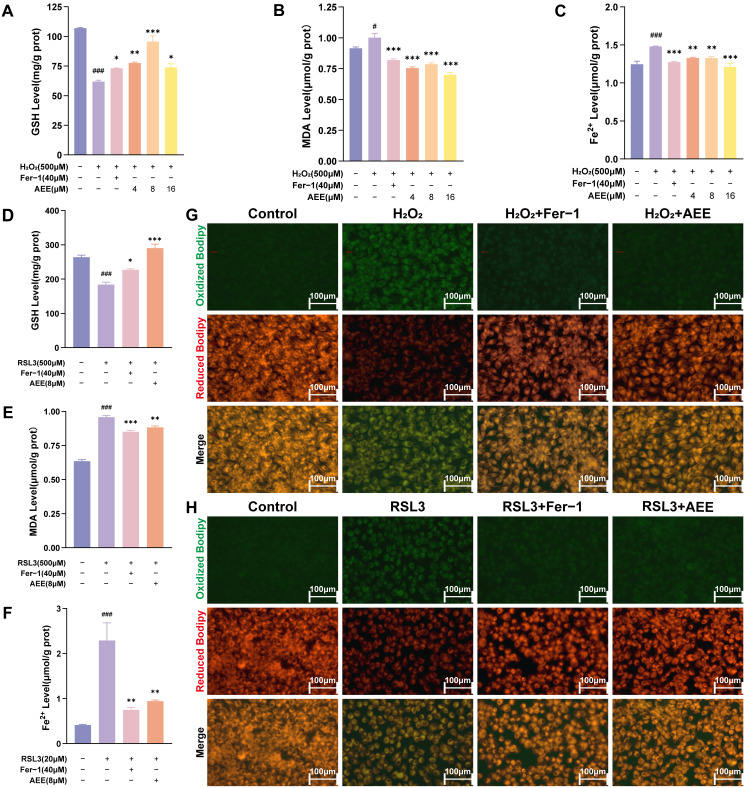
AEE inhibited H_2_O_2_ or RSL3-induced ferroptosis in BAECs. (**A**–**C**) The intracellular levels of GSH, MDA, and Fe^2+^ of BAECs in each group under H_2_O_2_-induced (*n* = 3). (**D**–**F**) The intracellular levels of GSH, MDA, and Fe^2+^ of BAECs in each group under RSL3-induced (*n* = 3). (**G**,**H**) Intracellular LPO levels of BAECs in each group, under H_2_O_2_ or RSL3-induced, were visualized via 40× fluorescence microscopy, (Scale bar = 100 μm). Data are represented as mean ± SD. # *p* < 0.05, ### *p* < 0.001 versus the Control group; * *p* < 0.05, ** *p* < 0.01, *** *p* < 0.001 versus the H_2_O_2_ or RSL3 group.

As shown in Figure 2A–C, compared with the Control group, H_2_O_2_ significantly decreased intracellular GSH levels and increased MDA and Fe^2+^ levels in BAECs (*p* < 0.05). AEE or Fer-1 significantly reversed the H_2_O_2_-induced intracellular GSH, MDA, and Fe^2+^ level changes (*p* < 0.05). AEE (8μM) exhibited a favorable effect across all indicators, and so was selected for subsequent experiments. From Figure 2D–F, RSL3 significantly decreased intracellular GSH levels and elevated MDA and Fe^2+^ levels in BAECs (*p* < 0.05). AEE or Fer-1 also significantly inhibited these RSL3-induced related changes (*p* < 0.05). H_2_O_2_ or RSL3 significantly increased intracellular LPO levels in BAECs (*p* < 0.05). Notably, LPO levels in the AEE and Fer-1 groups were significantly lower than those in the corresponding H_2_O_2_ or RSL3 groups (*p* < 0.05) (Figure 2G,H and Figure S2).

### 3.3. AEE Enhanced Antioxidant Gene Expression and Inhibited Ferroptosis-Related Gene Expression in BAECs

**Figure 3:**
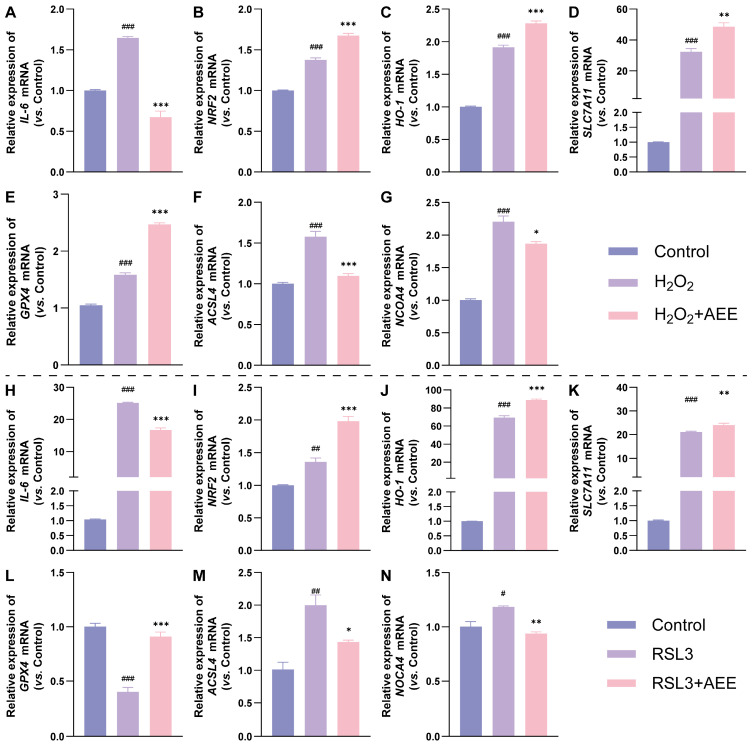
Effects of AEE on mRNA Levels in BAECs treated by RSL3 or H_2_O_2_ (*n* = 3). (**A**–**N**) The relative expression of *IL-6*, *NRF2*, *HO-1*, *SLC7A11*, *GPX4*, *ACSL4*, and *NCOA4* mRNA of BAECs in each group, under H_2_O_2_ (**A**–**G**) or RSL3-induced (**H**–**N**). Data are represented as mean ± SD. # *p* < 0.05, ## *p* < 0.01, ### *p* < 0.001 versus the Control group; * *p* < 0.05, ** *p* < 0.01, *** *p* < 0.001 versus the H_2_O_2_ or RSL3 group.

From Figure 3A–G, compared with Control group, the mRNA levels of *IL-6*, *NRF2*, *HO-1*, *SLC7A11*, *GPX4*, *ACSL4*, and *NCOA4* in H_2_O_2_ group were significantly upregulated (*p* < 0.05). Compared with H_2_O_2_ group, the mRNA levels of *NRF2*, *HO-1*, *SLC7A11*, and *GPX4* in H_2_O_2_+AEE group were significantly upregulated, while those of *IL-6*, *ACSL4*, and *NCOA4* were significantly downregulated (*p* < 0.05). As shown in Figure 3H–N, compared with the Control group, the mRNA levels of *IL-6*, *NRF2*, *HO-1*, *SLC7A11*, *ACSL4*, and *NCOA4* in the RSL3 group were significantly upregulated, while the mRNA level of *GPX4* was significantly downregulated (*p* < 0.05). Compared with the RSL3 group, the mRNA levels of *NRF2*, *HO-1*, *SLC7A11*, and *GPX4* in the RSL3+AEE group were significantly upregulated, while those of *IL-6*, *ACSL4*, and *NCOA4* were significantly downregulated (*p* < 0.05).

### 3.4. Transcriptome Analysis Results

**Figure 4:**
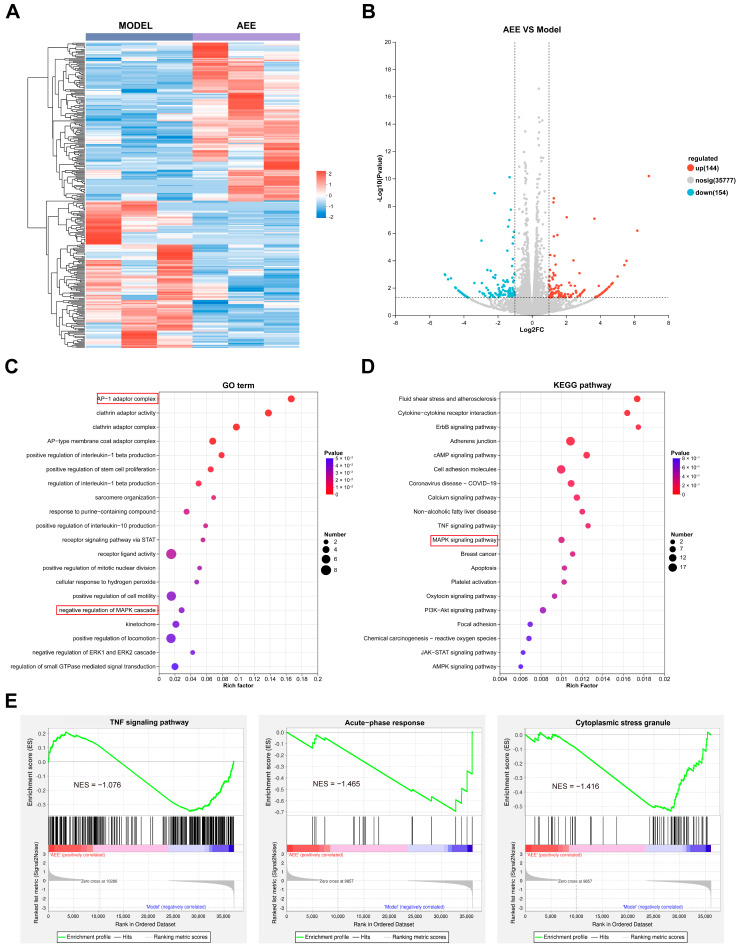
Transcriptomic analysis of AEE’s effects on H_2_O_2_-treated BAECs (*n* = 3). (**A**) Clustered Heatmap of DEGs between Model and AEE group. (**B**) Volcano plot of DEGs between Model and AEE group. (**C**) GO term enrichment analysis of DEGs. (**D**) KEGG pathways enrichment analysis of DEGs. (**E**) GSEA enrichment analysis.

Transcriptome sequencing was performed on BAECs in this study. As shown in Figure 4A,B, a total of 298 DEGs were identified between the model group and the AEE group. A total of 144 genes significantly upregulated and 154 genes significantly downregulated after AEE treatment, which may be related to the effect of AEE attenuating ferroptosis in BAECs. The GO enrichment analysis revealed 20 key biological processes that were significantly enriched by the upregulated and downregulated DEGs following AEE treatment, including the AP-1 adaptor complex, clathrin adaptor activity, clathrin adaptor complex, AP-type membrane coat adaptor complex positive regulation of interleukin-1 beta production, positive regulation of stem-cell proliferation, regulation of interleukin-1 beta production, sarcomere organization, the response to a purine-containing compound, positive regulation of interleukin-10 production, a receptor signaling pathway via STAT, receptor ligand activity, positive regulation of mitotic nuclear division, the cellular response to H_2_O_2_, positive regulation of cell motility, negative regulation of MAPK cascade, kinetochore, positive regulation of locomotion, negative regulation of the ERK1 and ERK2 cascade, and regulation of small GTPase mediated signal transduction (Figure 4C). The KEGG pathways enriched by the upregulated and downregulated DEGs following AEE treatment included fluid shear stress and atherosclerosis, cytokine–cytokine receptor interaction, ErbB signaling pathway, Adherens junction, cAMP signaling pathway, cell adhesion molecules, Coronavirus disease—COVID-19, calcium signaling pathway, non-alcoholic fatty liver disease, the TNF signaling pathway, the MAPK signaling pathway, breast cancer, apoptosis, platelet activation, oxytocin signaling pathway, the PI3K-Akt signaling pathway, focal adhesion, chemical carcinogenesis—reactive oxygen species, JAK-STAT signaling pathway, and the AMPK signaling pathway (Figure 4D). GSEA enrichment results indicated that compared with the Model group, the expression of gene sets related to the TNF signaling pathway and cytoplasmic stress granule pathway was downregulated in the AEE group (Figure 4E).

### 3.5. JNK Was a Key Protein Target for AEE Attenuating Ferroptosis

**Figure 5:**
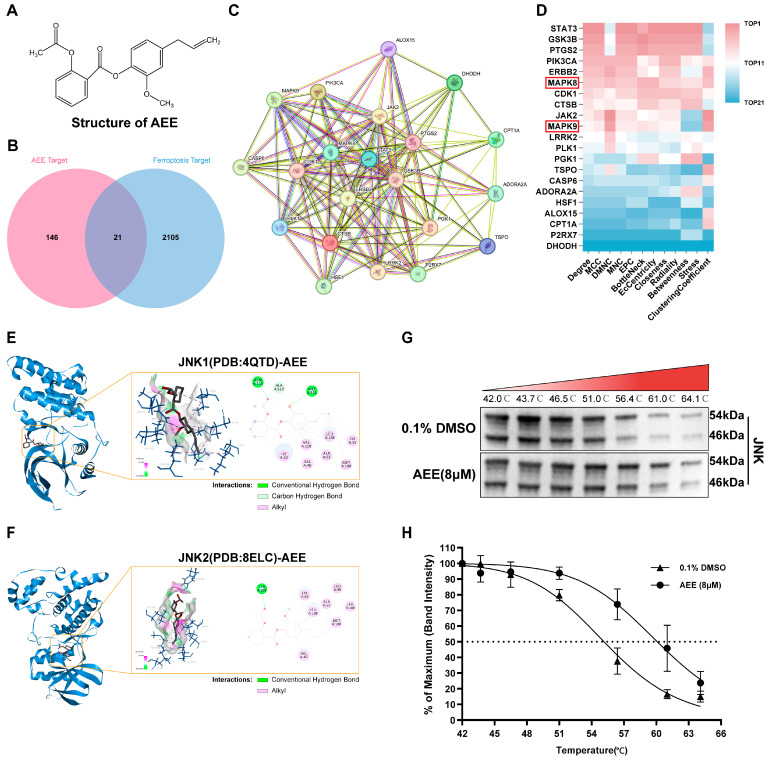
JNK was the key target protein of AEE. (**A**) A 2D structure of AEE. (**B**) Venn diagram of the intersection of AEE targets and ferroptosis targets. (**C**) PPI network analysis. (**D**) Ranking heatmap of key target scores analyzed by 12 algorithms. (**E**,**F**) Visualizations of molecular docking results for JNK1 and JNK2 with AEE. (**G**,**H**) Effect of AEE on the thermal stability of JNK (*n* = 3).

Transcriptome analysis suggested that the effect of AEE attenuating ferroptosis in BAECs might be associated with AP-1 complex formation downstream of MAPK, but the specific target proteins remained unclear. To identify direct target proteins of AEE, protein target prediction was performed, 167 potential targets of AEE were identified, and 2126 ferroptosis-related targets were retrieved from databases (Figure 5A–D). The Venn diagram was used to obtain 21 intersection genes, and a drug target-disease PPI network was constructed, containing 21 nodes and 122 edges. Twelve algorithms were applied to analyze core targets, and results showed that MAPK8 (JNK1) and MAPK9 (JNK2)—key proteins in the MAPK pathway—ranked top across all algorithm analyses and were identified as core targets. These two proteins were selected for further investigation.

Table 2 shows the statistical results of molecular docking between AEE or SP600125 and different PDB structures of JNK1 and JNK2. When the binding free energy (ΔG) between a compound and a protein is less than −7 kcal/mol, it can be considered that they have favorable binding affinity [35]. Molecular docking results indicated that AEE had favorable potential binding affinity to both JNK1 and JNK2. The binding modes of AEE or SP600125 with JNK1 and JNK2 are shown in Figure 5E,F and Figure S3. For JNK1 (PDB: 4QTD), the ΔG between AEE and JNK1 was −7.62 kcal/mol. AEE formed hydrogen bonds with the amino acid residues ASN114 and MET111 of JNK1, and hydrophobic interactions with ILE32, VAL40, VAL158, LEU168, ALA53, LYS55, and MET108. For JNK2 (PDB: 8ELC), the ΔG between AEE and JNK2 was −7.8 kcal/mol. AEE formed hydrogen bonds with the amino acid residues TYR185 of JNK2, and hydrophobic interactions with LEU88, LEU106, LEU168, LYS55, ALA53, MET108, and VAL40.

Based on the above results, CETSA between AEE and JNK were performed. Within the temperature range of 42–64.1 °C, AEE enhanced the thermal stability of JNK, with a shift in the thermal melting curve (Figure 5G,H). The aggregation temperature (Tagg) shifted from 55 °C to 60 °C.

### 3.6. H_2_O_2_ Induced Ferroautophagy via Activated JNK/c-Jun Phosphorylation

**Figure 6:**
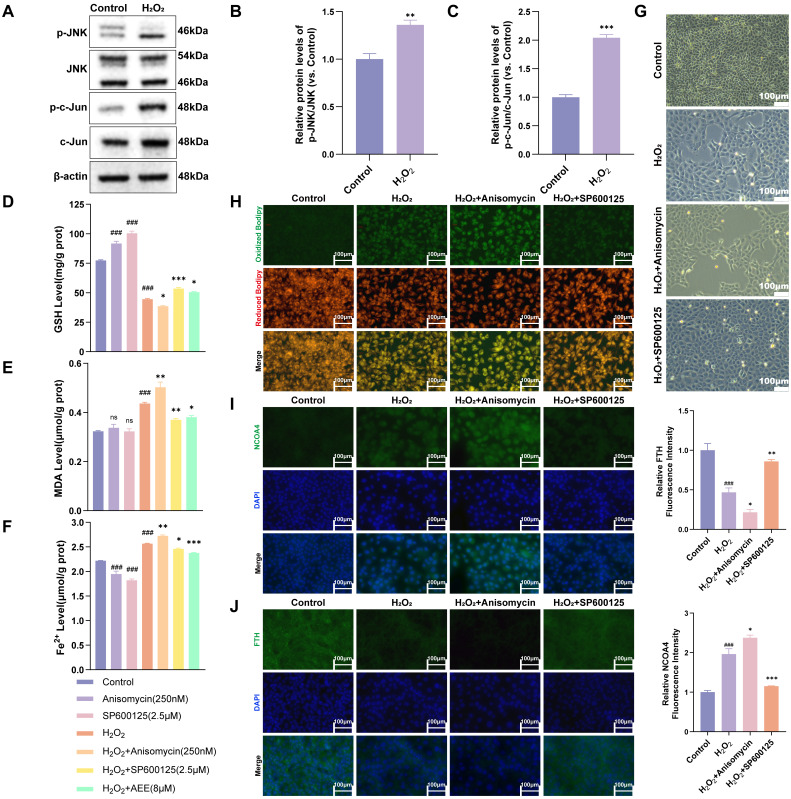
H_2_O_2_ induced ferritinophagy via activated JNK/c-Jun phosphorylation. (**A**–**C**) Phosphorylation level of JNK and c-Jun in BAECs induced with H_2_O_2_ (*n* = 3). (**D**–**F**) The intracellular levels of GSH, MDA, and Fe^2+^ of BAECs in each group (*n* = 3). (**G**) Effects of Anisomycin and SP600125 on H_2_O_2_-induced BAECs damage under 10× light microscopy, (Scale bar = 100 μm). (**H**) Effects of Anisomycin and SP600125 on LPO levels of BAECs induced with H_2_O_2_ (Scale bar = 100 μm). (**I**,**J**) Effect of Anisomycin and SP600125 on expression level of NCOA4 and FTH in BAECs induced with H_2_O_2_ (*n* = 3) (Scale bar = 100 μm). Data are represented as mean ± SD. ns *p* > 0.05, ### *p* < 0.001 versus the Control group; * *p* < 0.05, ** *p* < 0.01, *** *p* < 0.001 versus the H_2_O_2_ group.

To explore the effect of H_2_O_2_ on JNK/c-Jun activation, the phosphorylation levels of JNK/c-Jun in BAECs treated with H_2_O_2_ were measured. As shown in Figure 6A–C, H_2_O_2_ significantly increased the phosphorylation levels of JNK and c-Jun in BAECs (*p* < 0.05). Anisomycin significantly enhanced decreases in GSH levels, increases in MDA, Fe^2+^ and LPO levels, and cell detachment in H_2_O_2_-induced BAECs (*p* < 0.05). In contrast, SP600125 reversed these changes in GSH, MDA, Fe^2+,^ and LPO levels and cell morphology in H_2_O_2_-induced BAECs (*p* < 0.05) (Figure 6D–H and Figure S4). Anisomycin also further significantly promoted upregulation of NCOA4 and downregulation of FTH in H_2_O_2_-induced BAECs. In contrast, SP600125 suppressed upregulation of NCOA4 and downregulation of FTH in H_2_O_2_-induced BAECs (*p* < 0.05) (Figure 6I,J).

### 3.7. AEE Attenuates Ferroautophagy via Inhibiting JNK/c-Jun Phosphorylation in H_2_O_2_-Induced BAECs

**Figure 7:**
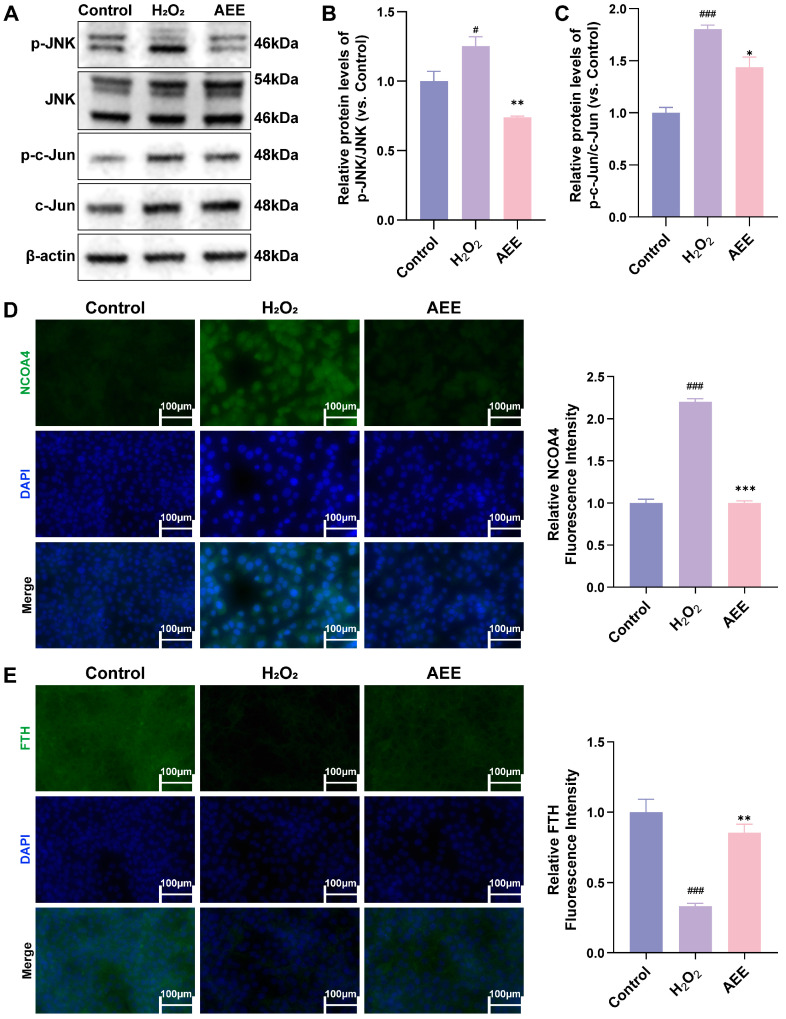
AEE attenuated ferroautophagy via inhibited JNK/c-Jun phosphorylation. (**A**–**C**) Effects of AEE on JNK and c-Jun phosphorylation level in BAECs induced with H_2_O_2_ (*n* = 3). (**D**,**E**) Effect of AEE on expression level of NCOA4 and FTH in BAECs induced with H_2_O_2_ (*n* = 3) (Scale bar = 100 μm). Data are represented as mean ± SD. # *p* < 0.05, ### *p* < 0.001 versus the Control group; * *p* < 0.05, ** *p* < 0.01, *** *p* < 0.001 versus the H_2_O_2_ group.

Compared with the Control group, the phosphorylation levels of JNK and c-Jun in BAECs were significantly increased in the H_2_O_2_ group (*p* < 0.05), while those in the AEE group were significantly lower than those in the H_2_O_2_ group (*p* < 0.05) (Figure 7A–C). H_2_O_2_ induced an increase in NCOA4 protein expression and a decrease in FTH protein expression in BAECs (*p* < 0.05). AEE significantly inhibited these H_2_O_2_-induced changes in NCOA4 and FTH protein expression (*p* < 0.05) (Figure 7D,E).

### 3.8. AEE Alleviates Endothelial Damage in a κ-Car-Induced Rat Aortic Vascular Endothelial Damage Model

**Figure 8:**
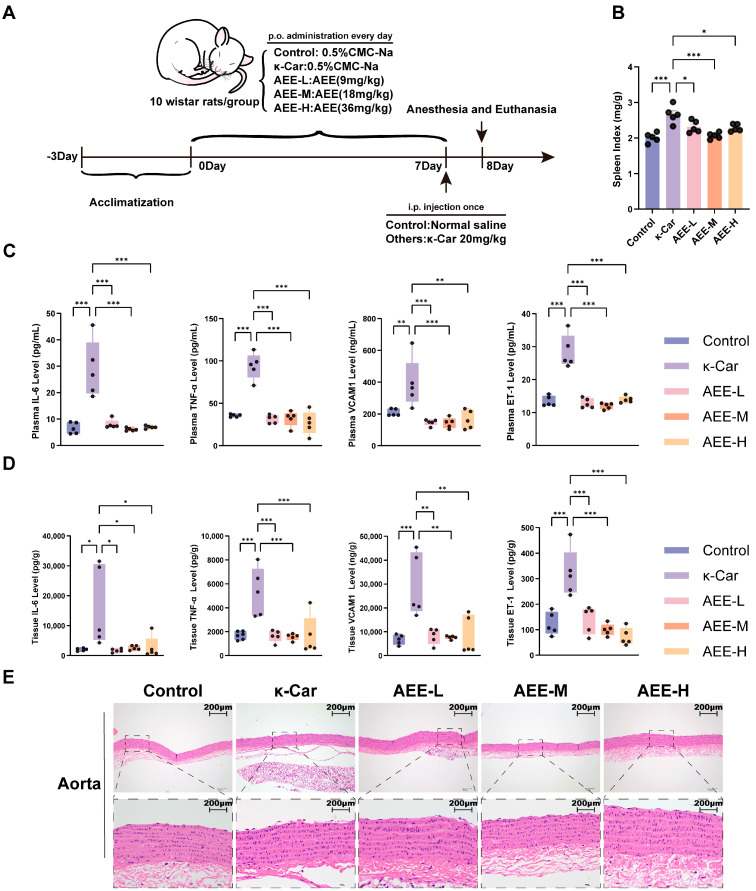
AEE attenuated κ-Car-induced vascular endothelial damage in rat aorta. (**A**) The establishment of a rat aortic vascular endothelial damage model and preventive administration protocol. (**B**) Rat spleen index (*n* = 5). (**C**) Levels of IL-6, TNF-α, VCAM1, and ET-1 in rat plasma (*n* = 5). (**D**) Levels of IL-6, TNF-α, VCAM1, and ET-1 in rat aorta (*n* = 5). (**E**) He staining of rat aorta (Scale bar = 200 μm). Data are represented as mean ± SD. Comparison among groups: * *p* < 0.05, ** *p* < 0.01, *** *p* < 0.001. Control: Control group, κ-Car: κ-Car group, AEE-L: AEE low-dose group, AEE-M: AEE medium-dose group, AEE-H: AEE high-dose group.

The workflow for establishing the κ-Car-induced rat aortic vascular endothelial damage model was shown in Figure 8A. Compared with the Control group, treatment of κ-Car significantly increased the spleen index and elevated the levels of inflammatory cytokines (TNF-α and IL-6), as well as endothelial damage markers (ET-1 and VCAM) in both plasma and aortic tissues (*p* < 0.05). The pretreatment of AEE significantly suppressed these κ-Car-induced increases in the spleen index, TNF-α, IL-6, ET-1, and VCAM levels (*p* < 0.05) (Figure 8B–D). HE staining results from the rat aorta revealed that in the κ-Car group, local endothelial cell nuclei in the rat aorta were exposed or endothelial cells detached into the lumen. The aortic endothelial morphology in all AEE groups was better preserved than in the κ-Car group (Figure 8E).

### 3.9. AEE Enhanced Antioxidant Capacity and Attenuated Ferroptosis in Rat Aorta

**Figure 9:**
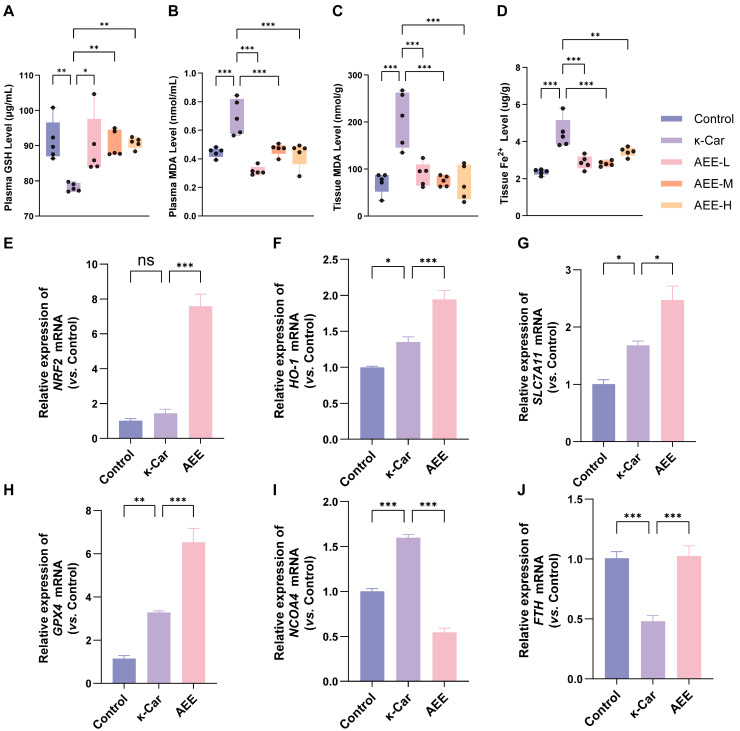
AEE enhanced antioxidant capacity and attenuated ferroptosis in rat aorta. (**A**–**D**) Levels of GSH, MDA, and Fe^2+^ in rat plasma or aorta (*n* = 5). (**E**–**J**) The relative expression of *NRF2*, *HO-1*, *SLC7A11*, *GPX4*, *NCOA4*, and *FTH* mRNA of rat aortic (*n* = 5). Data are represented as mean ± SD. Comparison among groups: ns *p* > 0.05, * *p* < 0.05, ** *p* < 0.01, *** *p* < 0.001. Control: Control group, κ-Car: κ-Car group, AEE-L: AEE low-dose group, AEE-M: AEE medium-dose group, AEE-H: AEE high-dose group.

Compared with the Control group, κ-Car significantly decreased GSH levels (in plasma) and increased MDA levels (in plasma and aorta) and Fe^2+^ levels (in aorta) (*p* < 0.05). Compared with the κ-Car group, κ-Car-induced relevant changes in rats were significantly inhibited in all AEE groups (Figure 9A–D). Based on the favorable performance of the AEE-M group in endothelial damage and ferroptosis indicators, this group was selected for subsequent experiments. After κ-Car treatment, the mRNA level of *NCOA4* in the aorta was significantly upregulated, whereas that of *FTH* was significantly downregulated (*p* < 0.05), while AEE significantly inhibited the change in these indicators (*p* < 0.05). Meanwhile, treatment of AEE further significantly enhanced κ-Car-induced upregulation in mRNA levels of *NRF2*, *HO-1*, *SLC7A11*, and *GPX4* (*p* < 0.05) (Figure 9E–J). These results indicated that AEE could enhance antioxidant capacity and alleviate κ-Car-induced rat aorta ferroptosis.

### 3.10. AEE Attenuates κ-Car-Induced Ferroautophagy of Rat Aorta via Inhibiting JNK/c-Jun Phosphorylation

**Figure 10:**
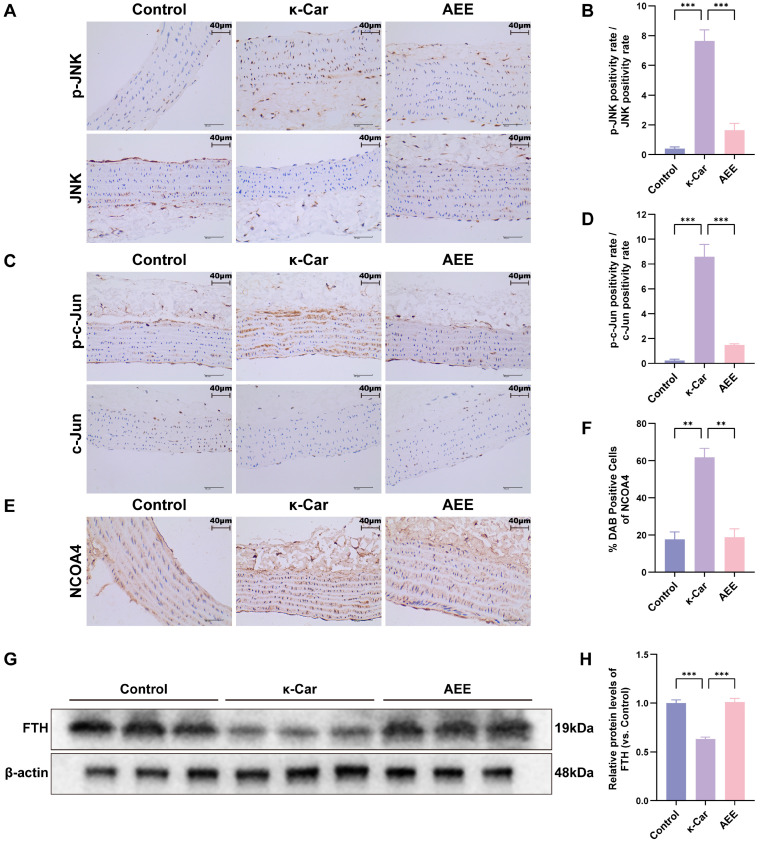
AEE attenuated κ-Car-Induced ferroautophagy of rat aorta via inhibited JNK/c-Jun phosphorylation. (**A**,**B**) Immunohistochemical staining images and ratio of positive cell rate for p-JNK and JNK expression in rat aorta (*n* = 5) (Scale bar = 40 μm). (**C**,**D**) Immunohistochemical staining images and ratio of positive cell rate for p-c-Jun and c-Jun expression in rat aorta (*n* = 5) (Scale bar = 40 μm). (**E**,**F**) Immunohistochemical staining images and positive cell rate of NCOA4 expression in rat aorta (*n* = 5) (Scale bar = 40 μm). (**G**,**H**) Relative expression levels of FTH in rat aorta (*n* = 3). Data are represented as mean ± SD. Comparison among groups:, ** *p* < 0.01, *** *p* < 0.001.

As shown in Figure 10A–H, κ-Car significantly increased in the phosphorylation of JNK and c-Jun and protein levels of NCOA4, as well as significantly decreasing in protein levels of FTH in the rat aorta (*p* < 0.05). Compared with the κ-Car group, the AEE group significantly downregulated phosphorylation of JNK and protein levels of c-Jun and NCOA4, while it upregulated protein levels of FTH in rat aorta (*p* < 0.05).

## 4. Discussion

Vascular endothelial damage can lead to the occurrence of multiple diseases and subsequently cause severe economic losses. Therefore, the integrity of bovine vascular endothelial function and structure is beneficial to cattle health, thereby improving farming efficiency [3,4,5]. Under the stimulation of various external factors, an oxidative stress microenvironment was prone to forming in the bovine. The occurrence of oxidative stress can induce pathological damage to VECs, subsequently leading to a decline in immune function, reduced production performance, and abnormally increased culling rate of bovine, ultimately resulting in severe economic losses [7,36]. Therefore, an in-depth analysis of the molecular mechanisms of oxidative stress-induced VEC damage in bovine and the development of targeted intervention drugs had important practical significance. Notably, the mechanisms of VEC damage induced by oxidative stress may involve ferroptosis—a programmed cell death mode driven by iron-dependent lipid peroxidation. Specifically, the occurrence of ferroptosis was inseparably linked to the disruption of the dynamic balance between lipid peroxidation and the elimination of lipid peroxidation products [37]. Previous studies confirmed that AEE could significantly alleviate oxidative stress-mediated VEC damage by enhancing cellular antioxidant capacity [38]. The enhancement of the cellular antioxidant effect by AEE suggested that it might have the potential to inhibit lipid peroxidation and thereby intervene in ferroptosis. The favorable performance of AEE in antioxidant capacity provided a new candidate drug for the prevention and treatment of oxidative stress-induced VEC damage in bovine.

When bovine was in an oxidative stress state, H_2_O_2_ was produced in large quantities in vivo. Excessive H_2_O_2_ caused vascular endothelial damage and the occurrence of ferroptosis [39,40,41]. RSL3 was a ferroptosis inducer that directly inhibited GPX4 activity, thereby blocking the clearance of LPO [26]. κ-Car, a potent pro-inflammatory substance, was commonly used to establish rat paw edema models and thrombosis models; it induced inflammation, oxidative stress, and ferroptosis of VECs via damaged VECs [42,43,44,45]. Therefore, this study constructed an in vitro ferroptosis model in BAECs, using H_2_O_2_ or RSL3 combined with an in vivo rat aortic endothelial damage model induced by κ-Car, to systematically reveal the molecular mechanisms of oxidative stress-induced vascular endothelial ferroptosis and validate the intervention effects of AEE. The results showed that H_2_O_2_ activated the JNK/c-Jun signaling pathway to upregulate NCOA4 expression, which further promoted the autophagic degradation of FTH, resulting in intracellular Fe^2+^ accumulation. This accumulated Fe^2+^ enhanced lipid peroxidation via the Fenton reaction, ultimately inducing ferroptosis in BAECs. In vivo experiments confirmed that κ-Car could induce vascular endothelial damage and ferroptosis in rats, primarily through the synergistic effect of enhancing oxidative stress and lipid peroxidation levels. Notably, AEE exhibited significant protective effects against vascular endothelial ferroptosis in both in vitro and in vivo models. Its mechanisms involved the following: (1) upregulating antioxidant gene expression in VECs to enhance lipid peroxide clearance capacity; and (2) inhibiting the activity of the JNK/c-Jun/NCOA4/FTH pathway to block ferritin autophagy-dependent iron overload. These dual mechanisms collectively suppressed the occurrence of vascular endothelial ferroptosis. These results elucidated the molecular pathways of oxidative stress-induced vascular endothelial ferroptosis and the specific mechanisms underlying the anti-ferroptosis effects of AEE from both in vitro and in vivo perspectives.

An imbalance in the lipid peroxidation metabolism drove ferroptosis in VECs [46,47]. The core regulatory mechanisms of this process involved two interrelated key processes: excessive generation of LPO and dysfunction of the antioxidant defense system, both of which are closely associated with oxidative stress. Under oxidative stress conditions [48,49,50,51], certain ROS, such as H_2_O_2_, reacted with Fe^2+^ via the Fenton reaction (H_2_O_2_ + Fe^2+^ → Fe^3+^ + ·OH OH^−^) to generate ·OH, which attacks PUFAs. This leads to the formation of phospholipid hydroperoxides (PLOOHs), initiating a lipid peroxidation chain reaction and causing LPO overload. This process was precisely regulated by key proteins, such as acyl-CoA synthetase 4 (ACSL4), which catalyzes the esterification of PUFA with coenzyme A, promoting their incorporation into membrane phospholipids and significantly increasing the sensitivity of membrane lipids to a ·OH attack. Additionally, NCOA4 mediated the autophagic degradation of FTH, promoted the release of stored iron as free Fe^2+^, and enhanced the Fenton reaction. On the other hand, oxidative stress depleted GSH and impaired the function of the SLC7A11/GPX4 antioxidant axis, jointly disrupting the capacity to eliminate lipid peroxidation products—impaired cystine uptake mediated by SLC7A11 leads to insufficient raw materials for GSH synthesis, while reduced GPX4 activity directly weakens its ability to use GSH to reduce and clear PLOOHs [49,52,53]. The abnormal accumulation of lipid peroxidation products and defects in their clearance capacity together triggered ferroptosis.

This study found that H_2_O_2_ could induce damage in BAECs, increased intracellular MDA and LPO levels, and decreased GSH levels, which was consistent with the typical characteristics of lipid peroxidation in ferroptosis. Pretreatment with the ferroptosis inhibitor Fer-1 [54] reversed H_2_O_2_-induced BAEC damage and abnormal change in related indicators, indicating that ferroptosis was a key pathway through which H_2_O_2_ mediates damage BAECs. Further experimental results showed that H_2_O_2_ upregulated the gene mRNA expression levels of *ACSL4* and *NCOA4*, and Fe^2+^ levels. The results indicated that H_2_O_2_-induced ferroptosis in BAECs was achieved by enhancing the driving of lipid peroxidation reactions. Notably, H_2_O_2_ stimulation did not inhibit the LPO clearance system; instead, it significantly upregulated the mRNA expression of genes such as *NRF2*, *HO-1*, *SLC7A11*, and *GPX4*, suggesting that BAECs attenuated the ferroptosis through a compensatory antioxidant response [49,55]. These results indicated that H_2_O_2_ primarily induces ferroptosis in BAECs by enhancing lipid peroxide generation rather than disrupting the clearance system of LPO.

In response to the inhibition strategy of ferroptosis, the current research focused on two major mechanisms: strengthening the antioxidant defense system and regulating iron metabolism homeostasis. The former enhanced the clearance capacity of LPO by either activating NRF2 nuclear translocation to upregulate downstream antioxidant gene expression, such as *HO-1*, *SLC7A11*, and *GPX4*, or directly boosting antioxidant gene expression, which is the mechanism underlying the effects of multiple drugs [56,57,58,59,60,61]. The latter maintained iron metabolism homeostasis by inhibiting NCOA4-mediated autophagic degradation of FTH to reduce free Fe^2+^ release, thereby exerting effects of anti-ferroptosis [62,63]. Previous studies demonstrated that AEE could activate NRF2 and enhance GPx activity, significantly improve cellular antioxidant capacity, and reduce ROS accumulation, thus attenuating oxidative stress. Based on the favorable pharmacological performance of AEE in mitigating oxidative stress, this study further explored its intervention effects on the ferroptosis of VECs. The results revealed [23,24] that AEE inhibited ferroptosis through two key mechanisms: (1) AEE activated the mRNA expression of genes in the *NRF2*/*HO-1* and *SLC7A11*/*GPX4* pathway to enhance clearance capacity of LPO, and (2) AEE inhibited the process of LPO production. These dual effects collectively alleviated ferroptosis. AEE exerts its anti-ferroptosis effects through these two key mechanisms, which were validated by similar results in a ferroptosis model of BAECs induced by RSL3 [64].

Based on the ameliorative effect of AEE on H_2_O_2_-induced ferroptosis and further investigating the specific molecular mechanisms by which AEE inhibited ferroptosis, transcriptomic sequencing was performed on BAECs ferroptosis models before and after AEE treatment. Enrichment analysis results indicated that the effect of AEE in alleviating inflammation and oxidative stress might be associated with its impact on mRNA expressions of relevant pathways. For the possibility of AEE alleviating ferroptosis, this study focused on the MAPK pathway (negative regulation of MAPK cascade) and AP-1 complex formation (AP-1 adaptor complex), both of which were affected by AEE pretreatment. These findings suggested that AEE might target the MAPK/AP-1 regulatory axis and inhibit lipid peroxidation and ferroptosis-related responses, thereby exerting endothelial protective effects.

The MAPK signaling pathway is closely associated with ferroptosis. Three MAPK family kinases–ERK, JNK, and p38–can influence the expression of ferroptosis-related proteins such as SLC7A11, GPX4, and NCOA4 by regulating downstream transcription factors, thereby participating in the ferroptosis process [65,66,67,68]. The AP-1 heterodimer, composed of c-Jun and c-Fos, belongs to a transcriptional activator regulated downstream of MAPK. MAPK can regulate AP-1 by influencing the phosphorylation of c-Jun and c-Fos, thereby participating in the occurrence of ferroptosis [69,70]. The JNK/c-Jun/NCOA4 axis is a critical pathway in the occurrence of ferroptosis [69]. After JNK is phosphorylated and activated, the activated JNK phosphorylates c-Jun in the AP-1 complex, enhancing the transcriptional activity of AP-1, which promotes the upregulation of NCOA4, leading to increased FTH degradation, elevated Fe^2+^ levels, cellular lipid peroxidation, and ultimately ferroptosis. Recently, there have been studies using compounds to promote or inhibit ferroptosis by influencing JNK/c-Jun phosphorylation [71,72,73,74]. Meanwhile, previous studies indicated that AEE clouds inhibit JNK phosphorylation in multiple models, suggesting that AEE may block the production of lipid peroxides by inhibiting JNK phosphorylation [24,38,75].

H_2_O_2_ activated JNK/c-Jun phosphorylation in BAECs. Pretreatment with a JNK phosphorylation agonist (Anisomycin) enhanced NCOA4 expression and ferritinophagy induced by H_2_O_2_, thereby promoting ferroptosis. Conversely, pretreatment with a JNK phosphorylation inhibitor (SP600125) suppressed the H_2_O_2_-induced ferritinophagy process. These results indicated that H_2_O_2_ induced ferroptosis by activating JNK phosphorylation to stimulate NCOA4 expression, leading to FTH degradation, elevated Fe^2+^ levels, and subsequent lipid peroxidation in BAECs. AEE directly affected JNK to inhibit its phosphorylation, downregulated NCOA4 expression, suppressed FTH degradation, and thereby inhibited ferroptosis caused by elevated Fe^2+^ levels. Therefore, AEE ameliorated ferroptosis in BAECs by inhibiting the JNK/c-Jun/NCOA4/FTH axis.

To validate the mechanisms by which AEE improves vascular endothelial damage and ferroptosis in vivo, this study established a rat aortic vascular endothelial damage model using intraperitoneal injection of κ-Car. The results showed that κ-Car-induced vascular endothelial damage led to elevated levels of endothelial damage factors, including VCAM1 and ET-1. Changes in GSH, MDA, and Fe^2+^ levels in the aorta indicated that κ-Car could induce oxidative stress and ferroptosis in rat aortic endothelial cells. The molecular mechanism underlying κ-Car-induced ferroptosis is similar to H_2_O_2_-induced ferroptosis in BAECs. Both processes act by activating the JNK/c-Jun/NCOA4/FTH axis to trigger ferritinophagy, ultimately leading to ferroptosis. Notably, as the H_2_O_2_-induced vitro model, κ-Car does not inhibit the clearance system of LPO, instead, it upregulated the mRNA expression of relevant anti-ferroptosis. On the other hand, AEE exhibited anti-inflammatory, anti-oxidative stress, and anti-ferroptosis pharmacological activities in a κ-Car-induced rat aortic vascular endothelial damage model. Consistent with its in vitro mechanism of anti-ferroptosis, AEE improves antioxidant capacity by enhancing the mRNA expression of *NRF2*/*HO-1* and *SLC7A11*/*GPX4*, and inhibits the ferritin ferritinophagy process mediated by the JNK/c-Jun/NCOA4/FTH axis. The regulation of these two aspects by AEE collectively exerts anti-ferroptosis effects. Notably, p53 is also a downstream target activated by JNK phosphorylation, and its activation inhibits SLC7A11 expression [70,76,77]. In both in vitro and in vivo experiments, AEE treatment further increased the mRNA expression of *SLC7A11* and enhanced antioxidant capacity. Although further studies on the effect of AEE on p53 activation were not conducted, the elevated SLC7A11 expression may be related to JNK phosphorylation inhibited by AEE, thereby further suppressing p53 activation. The regulatory mechanism of AEE on SLC7A11 needs further investigation. Due to limitations of current experimental conditions, this in vivo study on the alleviation of vascular endothelial damage by AEE used rats instead of bovine as experimental animals. The in vivo results from rat studies demonstrated that AEE enhanced antioxidant capacity in rat aortic vessels and suppressed ferritinophagy to inhibit ferroptosis, thereby mitigating vascular endothelial damage. These findings align with in vitro observations that AEE ameliorates vascular endothelial damage via boosting antioxidant defenses in BAECs and improving ferritinophagy by inhibiting the JNK/c-Jun/NCOA4/FTH axis to suppress ferroptosis. Based on these findings, in future, bovine will be used as clinical experimental animals to further validate the pharmacological efficacy and mechanisms of AEE in improving bovine vascular endothelial damage.

## 5. Conclusions

In summary, this study established H_2_O_2_-induced ferroptosis models in BAECs and κ-Car-induced ferroptosis models in rat aortic vascular endothelium, preliminarily investigating the mechanisms of ferroptosis induced by H_2_O_2_ and κ-Car. Through these in vitro and in vivo models, it was clarified that AEE alleviated ferroptosis through two key mechanisms: (1) AEE enhance the NRF2/HO-1 and SLC7A11/GPX4 axes to improve the clearance capacity of LPO in VECs. (2) AEE directly bind to JNK to inhibit its phosphorylation, thereby alleviating ferritin autophagy induced by the JNK/c-Jun/NCOA4/FTH axis. These two aspects collectively mitigate ferroptosis in VECs. The study on the specific mechanisms of AEE lays a foundation for providing candidate drugs for the prevention and treatment of bovine vascular endothelial damage.

## Figures and Tables

**Table 1:** Primer Information.

Gene Name	NCBI ID	Forward Primer (5′–3′)	Reverse Primer (5′–3′)
*GPX4* (Bovine)	NM_174770.4	TCCTCATTGACAAGAACGGCTGTG	TAGCACGGCAGGTCCTTCTCTATC
*NRF2* (Bovine)	NM_001011678.2	TCAGCCAGCACAACACATACCATC	ACGGGAATGTCTCTGCCAAAAGC
*HO-1* (Bovine)	NM_001014912.1	CCGCTACCTGGGAGACCTGTC	ACTTGGTGGCACTGGCGATATTG
*IL-6* (Bovine)	NM_173923.2	TGAGTGTGAAAGCAGCAAGGAGAC	CAAGCAAATCGCCTGATTGAACCC
*ACSL4* (Bovine)	XM_024988727.2	ATAGACATCCCTGGAGCAGACACC	TTATTCACTCGGCGGTTCACTTCG
*SLC7A11* (Bovine)	XM_024977578.2	TTCAAGGTGCCGCTGTTCATCC	TAGGCAGGAATCCCAGTCAGAGTG
*NCOA4* (Bovine)	NM_001075868.1	GGCTGCTGGAGTTGCTGATTC	TTCCTTGTTGGGACATCCTTCTTTG
*GAPDH* (Bovine)	NM_001034034.2	CGGCACAGTCAAGGCAGAGAAC	CCACATACTCAGCACCAGCATCAC
*GPX4* (Rat)	NM_174770.4	CCAGCAACAGCCACGAGTTCC	CACACGCAACCCCTGTACTTATCC
*NRF2* (Rat)	NM_001399173.1	TGCTCAACCGCTTGCTGTATGC	TCATCCGCCACTCATTCCTCTCC
*HO-1* (Rat)	NM_012580.2	CCGCCTTCCTGCTCAACATTG	TCTGTGAGGGACTCTGGTCTTTG
*IL-6* (Rat)	NM_012589.2	CTTCCAGCCAGTTGCCTTCTTG	TGGTCTGTTGTGGGTGGTATCC
*SLC7A11* (Rat)	NM_001107673.3	CTTTCAAGGTGCCTCTGTTCATCC	CAGTCAAGGTGATAAGGAAGCCAAC
*ACSL4* (Rat)	NM_001431649.1	TCACCATTGTATTGCTGCCTGTCC	CGGGTTTGTCTGAAGTGGGCTTAG
*NCOA4* (Rat)	NM_001034007.1	TGGCTCCTTAACAGTCATCAACAAG	AGAACTGGTGCTACAATGGCTATTAC
*FTH* (Rat)	NM_012848.2	TGCCATCAACCGCCAGATCAAC	AAGTTCTTCAGGGCCACATCATCC
*β-actin* (Rat)	NM_031144.3	GCTGTGCTATGTTGCCCTAGACTTC	GGAACCGCTCATTGCCGATAGTG

**Table 2:** Molecular docking results of AEE and SP600125 with multiple JNK1 and JNK2.

Compound		AEE		SP600125	
Protein	PDB	Binding Energy (kcal/mol)	pKi	Binding Energy (kcal/mol)	pKi
JNK1 (MAPK8)	4QTD	−7.62 ± 0.13	5.59 ± 0.10	−8.98 ± 0.13	6.60 ± 0.07
	4L7F	−7.58 ± 0.16	5.56 ± 0.12	−8.54 ± 0.21	6.26 ± 0.15
	3ELJ	−7.34 ± 0.23	5.38 ± 0.17	−8.98 ± 0.16	6.58 ± 0.12
	4AWI	−7.02 ± 0.13	5.23 ± 0.17	−7.92 ± 0.16	5.81 ± 0.12
JNK2 (MAPK9)	8ELC	−7.80 ± 0.12	5.72 ± 0.09	−8.34 ± 0.75	6.11 ± 0.55
	7N8T	−7.40 ± 0.16	5.42 ± 0.12	−9.10 ± 0.27	6.67 ± 0.20
	3NPC	−7.28 ± 0.08	5.34 ± 0.06	−8.26 ± 0.23	6.05 ± 0.17
	3E7O	−7.26 ± 0.11	5.32 ± 0.08	−8.84 ± 0.15	6.48 ± 0.11

Five parallel molecular docking results were used to calculate binding energy and pKi. Data are represented as mean ± SD.

## Data Availability

The data that support the findings of this study are available from the corresponding author upon reasonable request. Transcriptome data are available at NCBI databases under the accession number PRJNA1288194.

## Supplementary Materials

The following supporting information can be downloaded at: https://www.mdpi.com/article/10.3390/antiox14101220/s1. Figure S1: Cell viability of BAECs treated with different concentrations of Fer-1; Figure S2: Fluorescence ratios of oxidized Bodipy to reduced Bodipy in each group of BAECs induced with H_2_O_2_ or RSL3; Figure S3: Visualizations of molecular docking results for JNK1 and JNK2 with SP600125; Figure S4: Fluorescence ratios of oxidized Bodipy to reduced Bodipy in each group of BAECs treated with H_2_O_2_.

## Abbreviations

The following abbreviations are used in this manuscript:

VECs	Vascular Endothelial Cells
ROS	Reactive Oxygen Species
FTH	Ferritin Heavy Chain
NCOA4	Nuclear Receptor Coactivator 4
·OH	Hydroxyl Radical
PUFAs	Polyunsaturated Fatty Acids
Fe^2+^	Ferrous Ion
AEE	Aspirin Eugenol Ester
H_2_O_2_	Hydrogen Peroxide
HUVECs	Human Umbilical Vein Endothelial Cells
GSH	Glutathione
SOD	Superoxide Dismutase
MDA	Malondialdehyde
BAECs	Bovine Aortic Endothelial Cells
Fer-1	Ferrostatin-1
κ-Car	Carrageenan
CMC-Na	Sodium Carboxymethyl Cellulose
SPF	Specific Pathogen-Free
HE	Hematoxylin–Eosin
ELISA	Enzyme-Linked Immunosorbent Assay
VCAM1	Vascular Cell Adhesion Molecule 1
TNF-α	Tumor Necrosis Factor Alpha
IL-6	Interleukin 6
ET-1	Endothelin 1
FBS	Fetal Bovine Serum
LDH	Lactate Dehydrogenase
LPO	Lipid Peroxide
DEGs	Differentially Expressed Genes
GO	Gene Ontology
KEGG	Kyoto Encyclopedia of Genes and Genomes
PPI	Protein–Protein Interaction
PDB	Protein Data Bank
MAPK	Mitogen Activated Protein Kinase
JNK	c-Jun N-terminal Kinase
CETSA	Cellular Thermal Shift Assay
Tagg	Aggregation Temperature
NRF2	Nuclear Factor Erythroid 2-Related Factor 2
HO-1	Heme Oxygenase-1
SLC7A11	Solute Carrier Family 7 Member 11
GPX4	Glutathione Peroxidase 4
ACSL4	Long Chain Fatty Acid CoA Ligase 4
PLOOHs	Phospholipid Hydroperoxides
