# Supplementary material for: Aspirin Eugenol Ester Alleviates Vascular Endothelial Ferroptosis by Enhancing Antioxidant Ability and Inhibiting the JNK/c-Jun/NCOA4/FTH Signaling Pathway
Source: Antioxidants (Basel). 2025 Oct 10;14(10):1220. doi: 10.3390/antiox14101220 (PMC12561717; doi:10.3390/antiox14101220)
Supplement: Supplementary file 1 [file antioxidants-14-01220-s001.zip › antioxidants-3891403-supplementary.pdf]

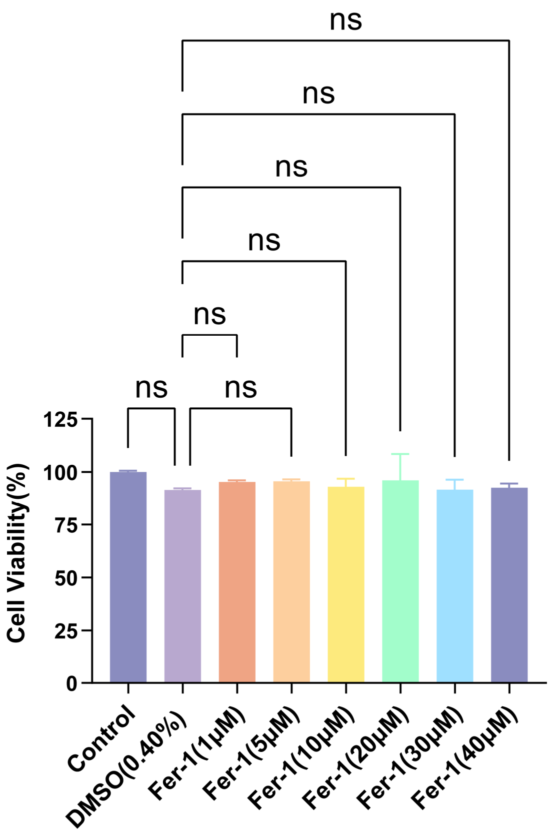

Figure S1 Cell viability of BAECs treated with different concentrations of Fer-1.

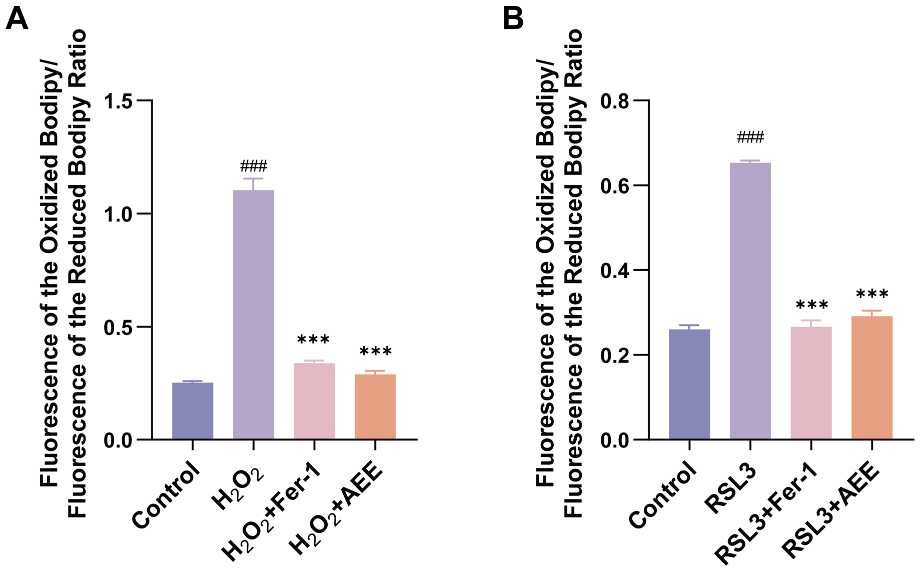

Figure S2 Fluorescence ratios of oxidized Bodipy to reduced Bodipy in each group of BAECs induced with H_2O_2 or RSL3. (A) Fluorescence ratios in each group treated with H_2O_2 as the inducer. (B) Fluorescence ratios in each group treated with RSL3 as the inducer. Data are represented as mean ± SD. ###*P* < 0.001 versus the Control group; ****P* < 0.001 versus the H_2O_2 or RSL3 group (n = 3).

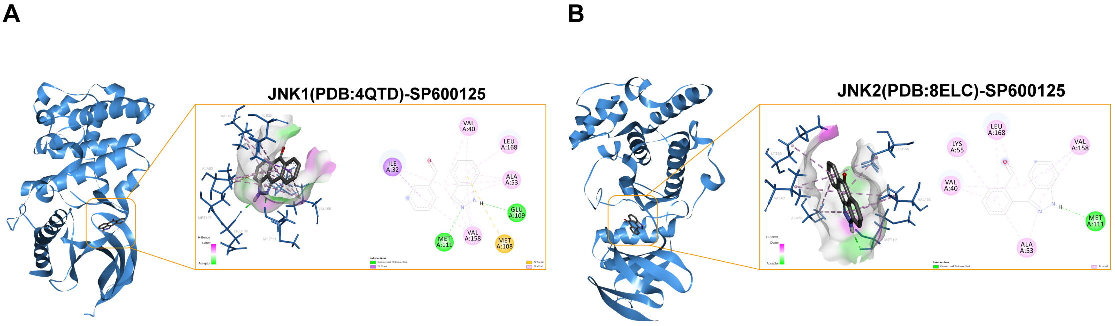

Figure S3 Visualizations of molecular docking results for JNK1 and JNK2 with SP600125.

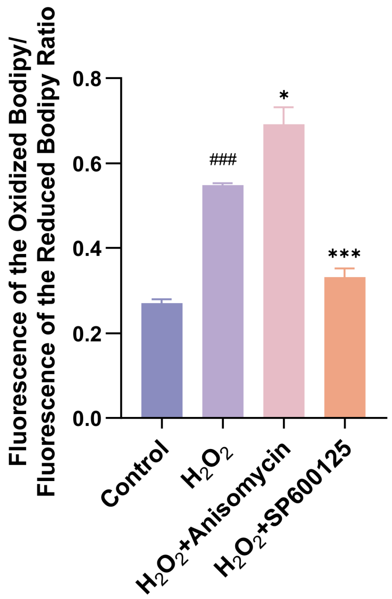

Figure S4 Fluorescence ratios of oxidized Bodipy to reduced Bodipy in each group of BAECs treated with H_2O_2. Data are represented as mean ± SD. ###*P* < 0.001 versus the Control group; **P* < 0.05, ****P* < 0.001 versus the H_2O_2 group (*n* = 3).
